# Equitable health care for people experiencing homelessness

**DOI:** 10.1016/j.eclinm.2023.102242

**Published:** 2023-09-19

**Authors:** eClinicalMedicine

Homelessness is an increasing global crisis that ranges from living in inadequate housing that is unsafe according to building regulations, to absolute homelessness, which includes sleeping in impromptu structures, in temporary or emergency shelters, or in the open. At least 150 million people worldwide are estimated to be homeless, and more than 1.8 billion do not have adequate housing. A multitude of systemic factors, such as poverty, unemployment, conflict, mental health disorders, natural disasters, and addiction are at the root of the problem. Although the specific causes of homelessness vary between and within countries, it mostly occurs when individual vulnerabilities and structural factors together lead to a breakdown of social support and an absence of income.

Health and homelessness are inextricably linked. People experiencing homelessness are at higher risk of developing chronic illnesses, such as cardiovascular disease, hepatitis, HIV, tuberculosis, and epilepsy, due to exposure to harsh environmental conditions, reduced access to nutrition and hydration, and barriers to regular health care. Simultaneously, untreated conditions, particularly mental health disorders and substance use problems, can propel people into homelessness by dismantling their financial stability, and these conditions can be made worse through periods of homelessness. Mental health disorders and substance use issues are disproportionately prevalent among homeless populations, and can be a result of traumatic experiences, unstable living conditions, or the absence of a support network. Furthermore, among people experiencing homelessness, mortality rates have been shown to be substantially higher than in the general population, with greater risk of premature mortality due to increased exposure to often inter-related risk factors, including substance misuse, smoking, unsafe sex, and violence.

For people experiencing homelessness, access to health care is often inadequate and can include numerous barriers, such as absence of personal identification and insurance, difficulties in registering with a primary care provider, and distrust in medical institutions. The absence of a fixed address can also lead to difficulties in accessing services, such as follow-up appointments and screening programmes. It has been shown that people experiencing homelessness might receive poor quality of care because of stigma and discrimination, which is likely to propagate the distrust in, and avoidance of, health-care providers. These barriers can result in more frequent emergency department visits, for health issues that could potentially have been avoided with earlier access to care and prevention.

In *eClinicalMedicine*, Tobias Schiffler and colleagues report their findings from a qualitative study that examined access to preventive cancer care for people experiencing homelessness across four countries in Europe. The authors found that specific cancer prevention programmes in this population were almost non-existent. In addition, health-care providers in some settings indicated that cancer in people experiencing homelessness was frequently not identified in the early stages and instead diagnosed only when the symptoms became more severe. These findings highlight the need for improvement in cancer screening and prevention in this vulnerable group and are discussed in more depth in our latest podcast. Additionally, in a systematic review published in our September issue, Vincy Chan and colleagues show that equity in clinical practice guidelines for homelessness and traumatic brain injury is suboptimal, and that there is a need for people experiencing homelessness to be more adequately considered in guideline development.

The unique health-care challenges faced by people experiencing homelessness require tailored interventions to overcome. Mobile clinics, outreach programmes, and shelters staffed with medical professionals have a crucial role in providing health care at proximity to people experiencing homelessness. Access to—and uptake of—self-care interventions, such as self-administered injectable contraception, self-monitoring of blood glucose for diabetes, and self-tests for HIV and pregnancy, must be improved among people experiencing homelessness in order to achieve better health outcomes. Addressing mental health and substance use challenges requires a multisectoral approach that combines psychiatry services, addiction therapy, and social support. Increased support through these approaches can also help build trust and prevent separation from the medical community.

In recent years, new methods have been suggested to close the gap between health-care providers and people experiencing homelessness. Medical respite programmes have been shown to be effective in improving outcomes for patients who are homeless after discharge from hospital. Additionally, standard case management, assertive community treatment, and critical time intervention have been shown to be effective models for mental health-care delivery in people experiencing homelessness. Models that involve collaborative care from medical professionals, social workers, and case managers ensure comprehensive support that extends beyond medical treatments and creates a continuum of care. The Health Navigator Model is being developed to improve cancer prevention access and awareness and reduce health inequalities experienced by this population. To overcome geographical barriers, telehealth platforms can also be used to connect people experiencing homelessness with health-care providers. Equity-oriented health care has been suggested as a framework for a more trauma-informed, equity-enhancing system, that is more accessible to people experiencing homelessness. Non-profit organisations and governments should foster partnerships to enable a holistic approach that addresses not only immediate health-care needs but also long-term pathways out of homelessness.

Although there have been some advances in interventions, more needs to be done. Substantial progress requires policy reform to prioritise people experiencing homelessness. Access to health care is a basic human right, and policy makers must endeavour to break down barriers such as restrictive identification requirements and difficulties in accessing insurance and registering with primary care providers. Increased funding for outreach programmes, community shelters, and clinics dedicated to people experiencing homeless will be crucial to improve health-care delivery in this population. Moreover, governments must seek to remedy the social policies and structural factors that result in homelessness. Urgent action is needed to make access to, and the provision of, health care for the homeless equitable with that for the general population.

